# Negative CT Contrast Agents for the Diagnosis of Malignant Osteosarcoma

**DOI:** 10.1002/advs.201901214

**Published:** 2019-10-11

**Authors:** Xianfu Meng, Hua Zhang, Meng Zhang, Baoming Wang, Yanyan Liu, Yan Wang, Xiangming Fang, Jiawen Zhang, Zhenwei Yao, Wenbo Bu

**Affiliations:** ^1^ Shanghai Key Laboratory of Green Chemistry and Chemical Processes College of Chemistry and Molecular Engineering East China Normal University Shanghai 200062 China; ^2^ Department of Radiology Huashan Hospital Fudan University Shanghai 200040 China; ^3^ State Key Laboratory of High Performance Ceramics and Superfine Microstructures Shanghai Institute of Ceramics Chinese Academy of Sciences Shanghai 200050 China; ^4^ School of Life Sciences University of Technology Sydney Sydney NSW 2007 Australia; ^5^ Department of Radiology Wuxi People's Hospital Nanjing Medical University Wuxi 214023 China

**Keywords:** H_2_ release, HMSN@AB@PEG, negative CT contrast agents, osteosarcoma, tumor acidic microenvironment

## Abstract

The current positive computed tomography (CT) contrast agents (PCTCAs) including clinical iodides, present high CT density value (CT‐DV). However, they are incapable for the accurate diagnosis of some diseases with high CT‐DV, such as osteosarcoma. Because bones and PCTCAs around osteosarcoma generate similar X‐ray attenuations. Here, an innovative strategy of negative CT contrast agents (NCTCAs) to reduce the CT‐DV of osteosarcoma is proposed, contributing to accurate detection of osteosarcoma. Hollow mesoporous silica nanoparticles, loading ammonia borane molecules and further modified by polyethylene glycol, are synthesized as NCTCAs for the diagnosis of osteosarcoma. The nanocomposites can produce H_2_ in situ at osteosarcoma areas by responding to the acidic microenvironment of osteosarcoma, resulting in nearly 20 times reduction of CT density in osteosarcoma. This helps form large CT density contrast between bones and osteosarcoma, and successfully achieves accurate diagnosis of osteosarcoma. Meanwhile, The NCTCAs strategy greatly expands the scope of CT application, and provides profound implications for the precise clinical diagnosis, treatment, and prognosis of diseases.

## Graphical abstract

A negative computed tomography (CT) contrast agent based on hollow mesoporous silica nanoparticles@ammonia borane@polyethylene glycol nanocomposite can produce H_2_ in situ at osteosarcoma areas by responding to the acidic microenvironment of osteosarcoma, and prominently reduce the CT density of osteosarcoma, which can form large CT density difference contrast between bones and osteosarcoma, and further realize accurate diagnosis of malignant osteosarcoma.

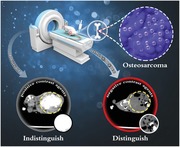

## 1 Introduction

As a prolific diagnostic imaging tool in clinic, X‐ray computed tomography (CT) is of great significance for detecting central nervous system disease,^1^ cardiovascular disease,^2^ and tumors^3^ due to its high time resolution, density resolution, and sensitivity.^4^ Since the attenuation coefficient of X‐ray is highly correlated with tissue density,^5^ the current positive CT contrast agents (PCTCAs) exhibit high CT density value (CT‐DV) and provide contrast enhancement in CT imaging.^6^ However, CT with PCTCAs fails to accurately detect diseases that have similar X‐ray attenuations to surrounding tissues, such as osteosarcoma, the most common primary bone malignancy in skeletal system disease.^7^ For example, enhanced CT scanning can barely distinguish osteosarcoma from surrounding bones in high energy level images. This is because bones and PCTCAs of high‐Z elements such as iodide, the lutecium‐based upconversion nanoparticles,^8^ can generate similar X‐ray attenuations and then result in analogous high CT density between them. Consequently, PCTCAs suffer from insufficient ability to distinguish osteosarcoma from bones. Rationally reducing the density of osteosarcoma can enhance the contrast between osteosarcoma and bone, which is propitious to detect osteosarcoma sensitively.

How can we reduce the density of osteosarcoma? A phenomenon in clinical CT lung inspection demonstrates the lung has lower CT density than the surrounding tissues. Briefly, in CT images, lung cavity with low CT density shows up as a dark region,^9^ while ribs of high CT density beside the lung present distinctly highlight signals. This is because the lung cavity full of gas has less X‐ray attenuation, on the contrary, the ribs possess higher X‐ray attenuation. This phenomenon inspires us that we can take advantage of gas to reduce CT density of osteosarcoma, further resulting in striking definition between osteosarcoma and bones, and finally achieve accurate osteosarcoma diagnosis.

**Scheme 1:**
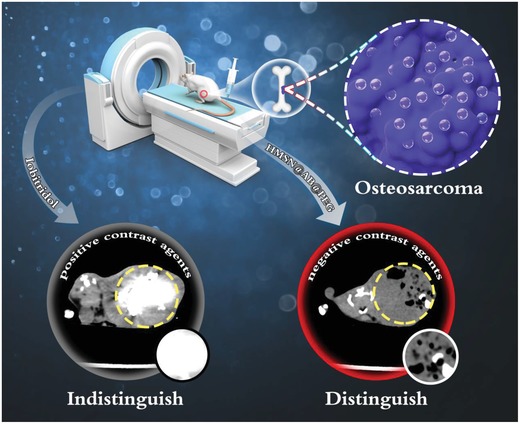
Schematic diagram of negative CT contrast agents for the accurate diagnosis of osteosarcoma, the area of dashed line is osteosarcoma. Apparently, negative CT contrast agents HMSN@AB@PEG could produce H_2_ to decrease the CT density value in the osteosarcoma area, further distinguishing osteosarcoma from bone, while the clinical CT contrast agent Iobitridol indistinguishes osteosarcoma from bone because of the similar X‐ray attenuations generated by bones and Iobitridol around osteosarcoma, both of which reveal high CT density.

**Figure 1:**
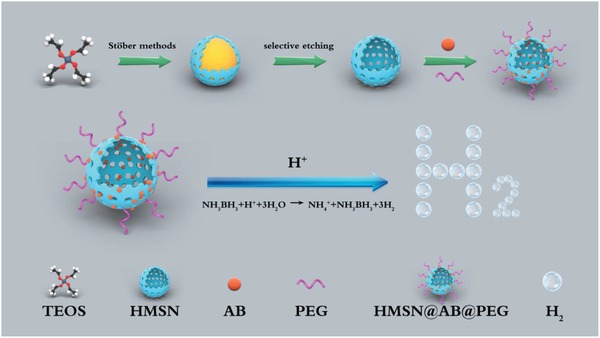
Diagram of HMSN@AB@PEG synthesis and H_2_ release mechanism.

Herein, we proposed a novel strategy of negative CT contrast agents (NCTCAs) for the precise diagnosis of solid tumors without a cavity, such as osteosarcoma. NCTCAs with lower CT density were capable of reducing the CT density value of target lesion areas, which made lesion areas exhibit dark signals in CT images, and further achieving accurate diagnosis. We designed a negative CT contrast agent HMSN@AB@PEG that produced gas in situ at the osteosarcoma site,^10^ and sharply reduced the CT density in tumors. A large density difference contrast formed between osteosarcoma and bone, and further resulted in accurate diagnosis of osteosarcoma (**Scheme** 1). Briefly, ammonia borane (AB) molecules, as H_2_ storage mediums, were utilized as H_2_ donors and efficiently loaded in hollow mesoporous silica nanoparticles (HMSN) by electrostatic adsorption and hydrogen bond interaction. Then, the nanocomposites could respond to the acid microenvironment of osteosarcoma and release H_2_. That was the reaction of NH_3_BH_3_ + H^+^ + 3H_2_O→NH_4_ + B(OH)_3_ + 3H_2_ (**Figure** 1). The product H_2_ could reduce the CT density of tumor area, resulting in remarkable contrast between osteosarcoma and bones, through which was conceivable to detect osteosarcoma precisely. Moreover, this strategy made it possible to detect solid tumors without a cavity using NCTCAs. The outcome of this study implied that the application scope of CT could be greatly expanded, providing profound implications for accurate clinical diagnosis, treatment, and prognosis of tumors and other diseases.

## 2 Results and Discussions

### 2.1 Synthesis and Characterization of HMSN@AB@PEG

**Figure 2:**
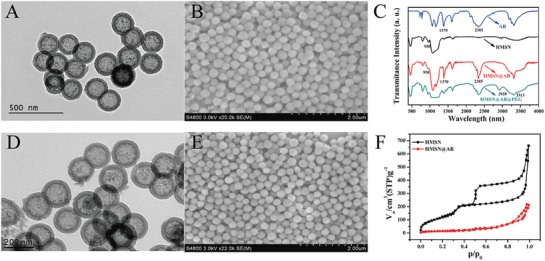
A,B) TEM and SEM images of HMSN; C) FTIR of AB, HMSN, HMSN@AB, and HMSN@AB@PEG; D,E) TEM and SEM images of HMSN@AB@PEG; F) N_2_ adsorption and desorption isothermal curves of HMSN and HMSN@AB.

Monodispersed HMSN nanoparticles were synthesized according to a classical selective etching strategy with some modifications.^11^ Briefly, dense solid SiO_2_ nanoparticles were first fabricated through a typical Stöber method (Figures S1A,B and S2A, Supporting Information). Mesoporous silica shells were then coated on the surface of solid SiO_2_ nanoparticles (Figures S1C,D and S2B, Supporting Information). Subsequently, the nanoparticles were selectively etched in Na_2_CO_3_ solution, and further extracted in NH_4_Cl solution to form the final HMSN nanoparticles. The obtained HMSN nanoparticles presented uniformly spherical feature (**Figure** 2A) with good hydrodynamic size distribution centered at 313.3 nm (Figure S3A, Supporting Information), which indicated that HMSN owned high dispersity without apparent aggregation. After loading with AB and PEG molecules (Figure 2D), the morphology of HMSN@AB@PEG still kept intact and the hydrodynamic size distribution centered at 339.5 nm (Figure S3B, Supporting Information). Meanwhile, the zeta potential of HMSN turned from −18.1 to −7.26 mV (HMSN@AB) after loading AB molecules (Figure S4, Supporting Information). Besides, scanning electron microscopy (SEM) images also verified the spherical and monodispersed morphology of as‐synthesized HMSN and HMSN@AB@PEG (Figure 2B,E).

AB as one of popular hydrogen storage materials^12^ has long been favored among hydrogen energy development.^13^ Herein, we utilized AB molecules as H_2_ gas donors to construct a kind of CT gas contrast agent to monitor osteosarcoma. Fourier transform infrared (FTIR) spectra exhibited AB molecules were successfully encapsulated into HMSN, as indicated by the overlap of characteristic peaks of HMSN and AB (Figure 2C). Subsequently, the N_2_ adsorption–desorption tests showed that HMSN had a larger specific surface area of 801.14 m^2^ g^−1^ (Figure 2F) and two pore sizes of 2.78 and 3.87 nm (Figure S5, Supporting Information). The different pore sizes may be attributed to alkaline Na_2_CO_3_ solution etching. After loading AB molecules, the specific surface area of HMSN dramatically decreased and the mesoporous properties disappeared, indicating the successful loading of AB. As shown in Figure S6 (Supporting Information), the thermogravimetric analysis (TGA) data presented about 20.7 wt% mass loss, which further demonstrated AB molecules were successfully married to HMSN nanoparticles.

### 2.2 H_2_ Release and Negative CT Contrast Performance in Aqueous Solutions of Different pH

**Figure 3:**
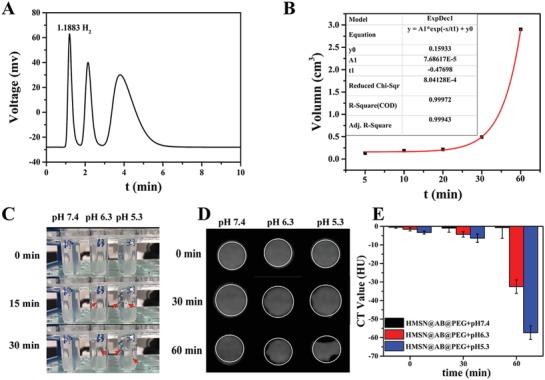
A,B) Gas chromatography of H_2_ release and dynamic curve of H_2_ release; C) H_2_ release images of HMSN@AB@PEG in different pH (5.3, 6.3, 7.4) after half hour, and the sites of the arrows are H_2_ bubbles; D) CT images of HMSN@AB@PEG in different pH (5.3, 6.3, 7.4). Obviously, CT value decreased in consequence of H_2_ release and the area of white line was the region of interest (ROI). E) Normalized CT value relative to water of the corresponding solutions, calculated by the average HU value of eight slices.

AB molecules could release H_2_ by responding to acid, followed by the reaction of NH_3_BH_3_ + H^+^ + 3H_2_O→NH_4_ + B(OH)_3_ + 3H_2_. So gas chromatography was utilized for qualitative and quantitative analysis of H_2_. At first step, a standard curve of different volume of H_2_ was drawn (Figure S7, Supporting Information). HMSN@AB@PEG nanoparticles were then dispersed in phosphate buffered saline (PBS) (pH 6.3) in a sealed container to react for 4 h. Subsequently, 1 mL microsyringe was used to assimilate the upper gas in the container and transferred the gas into the vaporizer immediately. Eventually, the H_2_ release volume from HMSN@AB@PEG was calculated as 2.4347 mL (**Figure** 3A), and the dynamic curve of H_2_ release was obtained as well (Figure 3B). According to the equation *pV* = *nRT*, the H_2_ mass was calculated to be about 0.196 mg. The H_2_ release process from free AB molecules and HMSN@AB@PEG in different pH (5.3, 6.3, 7.4) PBS was also recorded. Obviously, free AB molecules quickly turned into H_2_ in acidic conditions, in addition, the higher acidity could speed the H_2_ release process (Figure S8 and Video S1, Supporting Information). As a contrast, the similar phenomena was observed from HMSN@AB@PEG nanoparticles responding to acid (Figure S9 and Video S2, Supporting Information), which demonstrated HMSN@AB@PEG had the potential to release H_2_ as negative CT contrast agents in the acidic microenvironment of tumors. However, in PBS (pH 7.4), both of free AB molecules and HMSN@AB@PEG nanoparticles revealed no apparent AB decomposition/H_2_ release, indicating that HMSN@AB@PEG nanoparticles could not produce H_2_ in normal tissue environment.

We further investigated the CT contrast performance of HMSN@AB@PEG based on the above. First, H_2_ bubbles released in acidic solutions (pH 5.3, 6.3) instead of neutral pH solution (pH 7.4) (Figure 3C). Aqueous solutions of HMSN@AB@PEG in different pH (5.3, 6.3, 7.4) were then imaged using a CT scanner. Evidently, in acidic solutions, the region of interest (ROI) gradually decreased in CT images, on account of which H_2_ bubbles released to result in lower CT density. However, that of neutral pH solution changed little. Meanwhile, the normalized CT value of corresponding solutions relative to water was also calculated and summarized. The outcome implied higher acidity made more remarkable signals (Figure 3D,E). Even in a slight acid environment (pH 6.3) analogous to the acid microenvironment of tumors,^14^ the notable decrease of CT density value was also observed, demonstrating remarkable CT contrast performance of HMSN@AB@PEG. Moreover, the CT contrast performance of free AB molecules was also investigated, which showed analogous results with HMSN@AB@PEG (Figure S10, Supporting Information).

### 2.3 The Cellular Biocompatibility of HMSN@AB@PEG

To study the biocompatibility of HMSN@AB@PEG nanoparticles, 3‐(4, 5‐dimethylthiazol‐2‐yl)‐2, 5‐diphenyltetrazolium bromide (MTT) assay was implemented to evaluate the cellular cytotoxicity of HMSN@AB@PEG nanoparticles acting on osteosarcoma MG63 cells. There was no obvious cytotoxicity of HMSN@AB@PEG against the cells during a wide concentration range (0–100 ppm) (Figure S11, Supporting Information). Afterward, the phagocytosis of HMSN@AB@PEG nanoparticles by the cells was monitored by laser confocal fluorescence imaging (Figure S12A–C, Supporting Information). After incubated with HMSN@AB@PEG nanoparticles with fluorescein isothiocyanate (FITC) at 310 K for 4 h, the cells presented brightly green fluorescence, which demonstrated the cells could swallow the nanoparticles successfully. Moreover, the quantization line of FITC fluorescence intensity is shown in Figure S12D (Supporting Information), and the 3D video of FITC fluorescence intensity was also recorded, which exhibited higher FITC fluorescence intensity with high signal‐to‐noise ratio (Video S3, Supporting Information).

### 2.4 Negative CT Contrast Performance of HMSN@AB@PEG In Vivo

We next validated the CT imaging performance of HMSN@AB@PEG nanoparticles in vivo. First, we evaluated the osteosarcoma diagnosis efficiency by intratumor and intravenous injection of clinical CT contrast agent Iobitridol as PCTCAs. After intratumor injection of Iobitridol (350 000 ppm of I, 500 µL), the CT density of osteosarcoma area rapidly rose, presenting highlight signals in CT images (Figure S13, Supporting Information). However, we could not clearly distinguish the bone morphology from osteosarcoma. While intravenous injection of HMSN@AB@PEG (Figure S14, Supporting Information), we observed the similar results that we could not differentiate bone from tumor through both of gray and pseudo‐color images (transvers). We then evaluated the negative CT contrast performance of AB molecules in vivo (Figure S15, Supporting Information). Apparently, intratumor injection of AB molecules was capable of negative CT contrast performance soon. With the increase of hydrogen, CT density decreased gradually, which demonstrated AB molecules after intratumor injection enabled to produce H_2_ by responding to the tumor acidic microenvironment. In contrast, after intravenous of AB molecules, the CT density of tumor almost unchanged, which was attributed to the rapid metabolism of AB molecules in vivo and further led to ineffectively enrich in tumor.

**Figure 4:**
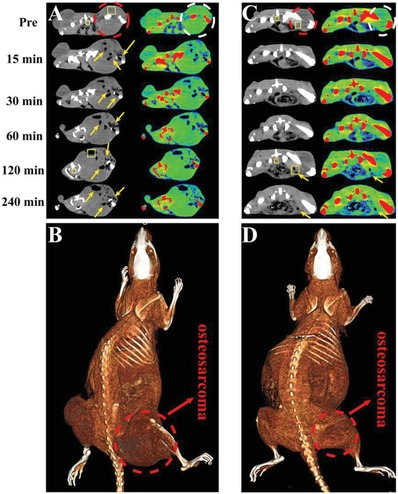
CT imaging of osteosarcoma‐bearing rats by intratumor and intravenous injection of HMSN@AB@PEG nanoparticles (at several time points). A,C) The intratumor and intravenous transvers gray images and corresponding pseudo‐color images were obtained. B,D) The corresponding 3D reconstruction maps of rat were obtained, intratumor and intravenous. The region of dashed line was the area of osteosarcoma, and the region of arrows was the low CT density area of H_2_ release. The two rectangle ROI in gray images (one was inside tumor, the other was in normal tissues) were selected to calculate the variation of CT density value between two time points (Pre and 120 min). FOV: 198 × 198 mm, *W*/*L*: 300/40.

After injection of HMSN@AB@PEG nanoparticles (30 000 ppm, 500 µL) into tumor, the CT density value of osteosarcoma obviously decreased along with the generation of H_2_ (**Figure** 4A). From gray and pseudo‐color images (transvers), we could accurately diagnose osteosarcoma no interference with bone of high CT density. The corresponding gray (Figure S16, Supporting Information) and pseudo‐color (Figure S17, Supporting Information) images (coronal) also indicated that a remarkable contrast emerged between bone with high CT density and osteosarcoma with low CT density. Furthermore, we measured the change of CT density value in rectangular ROI at two time points (Pre and 120 min) (Figure S18, Supporting Information). The CT density value of osteosarcoma significantly decreased, but the CT density value of normal tissues hardly changed. And the reduction extent of CT density was as high as 20 times. Besides, gray CT images (240 min) of different slices in the osteosarcoma were recorded as well (Video S4, Supporting Information). Finally, to further verify that gas did localize in the tumor area, digital image (Figure S19A, Supporting Information) and corresponding 3D reconstruction map (Figure 4B) of osteosarcoma‐bearing rat were supplemented. HMSN@AB@PEG was intratumorly injected into another osteosarcoma‐bearing rat 240 min later, CT imaging and magnetic resonance imaging (MRI) were conducted simultaneously. Obviously, CT density of the tumor decreased significantly (Figure S19B, Supporting Information). From the images of MRI (Figure S19C,D, Supporting Information), we could clearly find the location of osteosarcoma and there was much black phantom in the tumor, of which was caused by the produced H_2_ on account.

Next, we also evaluated the diagnosis performance of osteosarcoma after the intravenous injection of HMSN@AB@PEG. Analogously, as shown in Figure 4C,D, the reduced CT density value was clearly observed in tumor region compared with normal tissues after 2 h post‐injection. From the pseudo‐color images (transvers), we could easily discover the position of low CT density around the right leg of rat, indicating successful detection of osteosarcoma. Compared with AB molecules, this also demonstrated the important role of HMSN. The corresponding gray (Figure S20, Supporting Information) and pseudo‐color (Figure S21, Supporting Information) images (coronal) also clearly suggested that HMSN@AB@PEG as a negative CT contrast agent presented effective diagnosis of osteosarcoma. Furthermore, we calculated the change of CT density value in rectangular ROI at two time points (Pre and 120 min) before and after intravenous injection of HMSN@AB@PEG (Figure S22, Supporting Information). Similar to the intratumor injection, the CT density value of osteosarcoma reduced, while it hardly changed around normal tissues. The reduction extent of CT density was still about two times. From the above outcomes, we knew the strategy of NCTCAs for diagnosis of osteosarcoma was practicable.

### 2.5 The Biocompatibility In Vivo

Encouraged by the excellent negative CT contrast performance of HMSN@AB@PEG nanoparticles, the toxicity in vivo should be further studied. 100 µL of HMSN@AB@PEG solution (30 000 ppm) or PBS (as control) was intravenously injected into ICR mice to observe the variation of weight and physiological actions for 3 and 30 d. The bodyweight kept almost the same between both the groups (Figure S23, Supporting Information), at the same time, the results of serum biochemistry and hematology also exhibited little difference (Figure S24, Supporting Information). The hematoxylin and eosin (H&E) staining analysis of organs including heart, liver, spleen, lung, and kidney also showed similar morphologies between experiment groups and control groups (Figure S25, Supporting Information), further demonstrating the excellent biocompatibility and negligible side effects of HMSN@AB@PEG, which was suitable as negative CT contrast agent. Finally, we investigated the distribution of HMSN@AB@PEG in each organ. After 4 h intravenous injection of HMSN@AB@PEG, we found most of HMSN@AB@PEG were distributed in the liver and spleen, a few were in tumor (Figure S26, Supporting Information). And the H&E staining image of tumor was supplemented to verify the existence of tumor (Figure S27, Supporting Information).

## 3 Conclusion

In summary, we achieved successful detection of osteosarcoma through NCTCAs strategy. HMSN@AB@PEG nanoprobes as NCTCAs could specifically respond to the acidic microenvironment of tumors, and finally achieve sensitive detection of osteosarcoma. HMSN@AB@PEG nanoprobes had excellent biocompatibility and low toxicity. In vitro and in vivo experiments showed that HMSN@AB@PEG could rapidly generate H_2_ gas in the tumor area and then significantly reduce the CT density value in tumor region. The reduction extent of CT density was nearly as high as 20 times after intratumor injection of HMSN@AB@PEG. This helped form a sharp contrast between bones and osteosarcoma, and further realize accurate diagnosis of osteosarcoma. The strategy of NCTCAs that included gas or materials of low CT density was able to realize the precise diagnosis of diseases with high CT density, such as osteosarcoma, meanwhile, to provide a new idea for the detection of solid tumors without a cavity. Altogether, the strategy of NCTCAs could overwhelmingly promote the scope of CT application and provide profound implications for the diagnosis, treatment, and prognosis of tumors and other diseases in clinic.

## 4 Experimental Section

*Materials*: Cetyltrimethylammonium chloride (CTAC), triethanolamine (TEA), and tetraethyl orthosilicate (TEOS) were purchased from Sigma‐Aldrich; phosphate buffer solution was prepared by Na_2_HPO_4_, KH_2_PO_4_, hydrochloric acid, and deionized water. Na_2_HPO_4_, KH_2_PO_4_, AB, and Na_2_CO_3_ were acquired from Tansoole. Amino‐polyethylene glycol with molecular weight 2000 was purchased from Shanghai ToYong Biotechnology Co., Ltd. The NH_4_Cl, hydrochloric acid, and ethanol were purchased from Sinopharm Chemical Reagent Co., Ltd. All reagents were analytical without any purification unless otherwise specified.

*Characteristics*: Transmission electron microscopy (TEM) morphology was recorded on a JEOL JEM‐2100 at 200 kV (JEOL Ltd., Japan). SEM images were carried on a Hitachi S‐48000 scanning electron microscope. Dynamic light scattering (DLS) tests were conducted on a Malvern ZetasizerNano ZS analyzer. FTIR spectra were collected on a TENSOR II FT‐IR spectrophotometer (Bruker Corporation) by KBr. The concentration of materials and elements were quantified by an inductive coupled plasma (ICP) emission spectrometer (Agilent Technologies 5100 ICP‐OES). Gas chromatography was measured by a GC2060 gas chromatography system. The videos of gas releasing were acquired by an Apple Phone 7+. The CT measurements were obtained by a clinical CT instrument (GE discovery CT750 HD).

*Preparation of Hollow Mesoporous SiO_2_ Particles*: First of all, monodispersed solid SiO_2_ nanoparticles were prepared referring to the typical Stöber methods.^11^ Briefly, ultrapure water (10 mL), alcohol (74 mL), and ammonia solution (33% wt) (3.14 mL) were mixed homogeneously and stirred for 5 min in a 303 K water bath. Then 6 mL of TEOS was followed adding into the solution. After the above mixture was stirred for 1 h, solid SiO_2_ nanoparticles were obtained and washed with ethanol three times, then dispersed again in 60 mL deionized water. Second, 2 g CTAC and 0.4 g TEA were together added into 90 mL deionized water and stirred for 1.5 h to create a homogeneous phase. Then SiO_2_ (30 mL) aqueous solution was added and stirred for another 1.5 h. TEOS (1.8 mL) was added into the above solution dropwise and the mixture continued to be stirred for 1 h in a 353 K water bath. The resulting SiO_2_@SiO_2_ particles were centrifuged and washed three times by ethanol, and dispersed again in 30 mL of deionized water. Afterward, 18 mmol of Na_2_CO_3_ was added into the above solution and the system was stirred for 1 h at 353 K. The obtained nanoparticles were centrifuged and washed three times by deionized water. Eventually, CTAC was extracted by a rapid ion exchange method. Briefly, the above particles were dispersed in ethanol (60 mL) containing 3 g of NH_4_Cl and the system was further stirred at 333 K for 4 h.^15^ The resulting nanoparticles (HMSN) were centrifuged and washed three times by ethanol, and then dispersed in ethanol (80 mL).

*Preparation of HMSN@AB@PEG*: First of all, AB molecules (150 mg) were dissolved in HMSN aqueous solution (1 mL, 30 mg mL^−1^) completely, and then the system was stirred for 24 h at room temperature. AB molecules were trapped into the HMSN cavities and mesoporous channels through electrostatic adsorption and hydrogen bonding interaction. Afterward, HMSN@AB nanoparticles were collected by centrifugation and washed by deionized water. The resulting nanoparticles were dried via vacuum freeze‐drying for further use.

Then 100 mg of HMSN@AB nanoparticles were redispersed in 10 mL of ultrapure water. NH_2_‐PEG‐2000 molecules (500 mg) were added into the solution, and the system was continuously stirring for another 24 h. At last, HMSN@AB@PEG nanoparticles were collected by centrifugation, washed by deionized water, and dried via vacuum freeze‐drying.

*Cells Toxicity Assessment of HMSN@AB@PEG*: MG63 osteosarcoma cells were seeded in Dulbecco modified Eagle's medium (DMEM) with 9% fetal bovine serum (FBS) and 1% penicillin/streptomycin at 310 K for 24 h and with 5% CO_2_ with a density of 10^4^/well.^16^ Different concentrations (100, 50, 25, 12.5, 6.25 ppm) of HMSN@AB@PEG particles were added to the wells and coincubated for another 24 h. Subsequently, cell viability was tested by a typical MTT assay. Typically, 100 µL of MTT (0.6 mg mL^−1^) was added in each well and coincubated for 4 h, and the medium was then replaced by 100 µL of DMSO into per well. Several minutes later, the absorbance of the medium was monitored by a microplate reader (Bio‐TekELx800) at the 490 nm wavelength.

*The Confocal Fluorescence Images to Observe the Cellular Uptake of HMSN@AB@PEG*: To monitor the cellular uptake of HMSN@AB@PEG, HMSN@AB@PEG was modified by FITC. Briefly, FITC (1 mg) was dissolved in 10 mg mL^−1^ of HMSN@AB@PEG aqueous solution, and the system was stirred for 24 h. The final nanoparticles were centrifuged and washed three times by deionized water, and then redispersed in DMEM with 2% FBS as follows. 5 × 10^3^ MG63 osteosarcoma cells were cultured with DMEM containing 2% FBS in a coverglass dish at 310 K for 24 h and with 5% CO_2_. Subsequently, 100 ppm of HMSN‐FITC were added into the media and incubated for 4 h, and then the media was washed three times with physiological saline solution (PBS) for removing the free HMSN‐FITC. Finally, PBS (1 mL) was added into the media and the confocal fluorescence imaging experiments were carried out by using Nikon Confocal Microscope A1R+‐980 (Nikon Corporate, Japan).

*In Vivo Toxicity Evaluation of HMSN@AB@PEG*: Healthy Institute of Cancer Research (ICR) mice (≈35 g) were purchased and divided into three groups. 100 µL of HMSN@AB@PEG (30 000 ppm) was intravenously injected into ICR mice of experiment groups. As a contrast, 100 µL of PBS was injected into the control group. The experimental groups were sacrificed to study the short and long‐term toxicity of HMSN@AB@PEG at 3 and 30 d. The dissected tissues comprising heart, liver, spleen, lung, and kidney were H&E stained for histological analysis. Besides, the blood of mice was extracted for the toxicity investigation. All animal experiments were performed according to the guidelines of the Regional Ethics Committee for Animal Experiments, and the care regulations authorized by the Administrative Committee of Laboratory Animals of East China Normal University (accreditation number: m+ R20190701).

*Percentage Distribution of Si Element in Various Organs and Blood*: 100 µL of HMSN@AB@PEG (30 000 ppm) was intravenously injected into three ICR mice. After 4 h, the mice were sacrificed to obtain the dissected tissues comprising heart, liver, spleen, lung, kidney, and tumor. The dissected tissues were further dispersed in nitrohydrochloric acid. Two weeks later, the Si element was quantified according to ICP.

*Investigation of AB Loading Capacity of HMSN*: The AB loading capacity of HMSN was evaluated by TGA. First, almost 5 mg of HMSN@AB nanoparticles were dried via vacuum freeze‐drying overnight. TG test was implemented through temperature procedure from 298 to 1073 K, 10 K min^−1^. Finally, the TG curve was obtained, by which the AB loading capacity of HMSN was calculated to be 20.7%.

*Checkout of H_2_ Release from HMSN@AB@PEG Nanoparticles in pH 6.3 PBS*: First of all, gas chromatography was utilized to draw a standard curve of different volume of H_2_. Subsequently, 2.5 mg of HMSN@AB@PEG nanoparticles were dispersed in PBS (pH 6.3) and reacted for 4 h. Then one injection syringe was used to suck up the above gas (1 mL) in the container and injected into the vaporizer with carrier Ar gas. Eventually, H_2_ release volume from HMSN@AB@PEG was calculated as 2.4347 mL. According to the equation *pV* = *nRT*, H_2_ mass was about 0.196 mg.

*CT Intensity of Different Solutions*: All CT tests were performed on a clinical CT machine GE discovery CT750 HD, GE Healthcare in Huashan Hospital Affiliated to Fudan University. Several aqueous solutions containing HMSN@AB@PEG in different pH were investigated. And the test condition were abdomen, slice thick speed 0.625 mm. All aqueous solutions were put in 2 mL tube, and the final signal intensity was the average CT value of all slices.

*Construction of MG63 Tumor‐Bearing SD Rat Model*: The SD rats of about 300 g were purchased and raised at least two weeks before experiments. Then 200 µL of DMEM containing MG63 cells (10^8^) was injected near fibula and tibia. One week later, another same injection was performed to make sure the model was successful.

*CT Imaging In Vivo*: CT images in vivo were performed with conventional CT scanning mode. CT images of MG63 tumor‐bearing SD rats were conducted in different time periods after the intratumor and intravenous injection of 500 µL HMSN@AB@PEG solution (30 000 ppm). The tube voltage was 120 kVp, slice thickness was 0.625 mm.

## Conflict of Interest

The authors declare no conflict of interest.

## Supporting information

Supplementary

Supplementary

Supplementary

Supplementary

Supplementary
