# Supplementary material for: Negative CT Contrast Agents for the Diagnosis of Malignant Osteosarcoma
Source: Adv Sci (Weinh). 2019 Oct 11;6(23):1901214. doi: 10.1002/advs.201901214 (PMC6891899; doi:10.1002/advs.201901214)
Supplement: Supplementary file 1 — Supplementary [file ADVS-6-1901214-s002.pdf]

Copyright WILEY-VCH Verlag GmbH & Co. KGaA, 69469 Weinheim, Germany, 2019.

ADVANCED SCIENCE
Open Access

Supporting Information

for *Adv. Sci.*, DOI: 10.1002/advs.201901214

Negative CT Contrast Agents for the Diagnosis of Malignant Osteosarcoma

Xianfu Meng, Hua Zhang, Meng Zhang, Baoming Wang, Yanyan Liu, Yan Wang, Xiangming Fang, Jiawen Zhang, Zhenwei Yao,* and Wenbo Bu**

Copyright WILEY-VCH Verlag GmbH & Co. KGaA, 69469 Weinheim, Germany, 2019.

Supporting Information

Negative CT contrast agents for the diagnosis of malignant osteosarcoma

Xianfu Meng, Hua Zhang, Meng Zhang, Baoming Wang, Yanyan Liu, Yan Wang, Xiangming Fang, Jiawen Zhang, Zhenwei Yao*, Wenbo Bu**

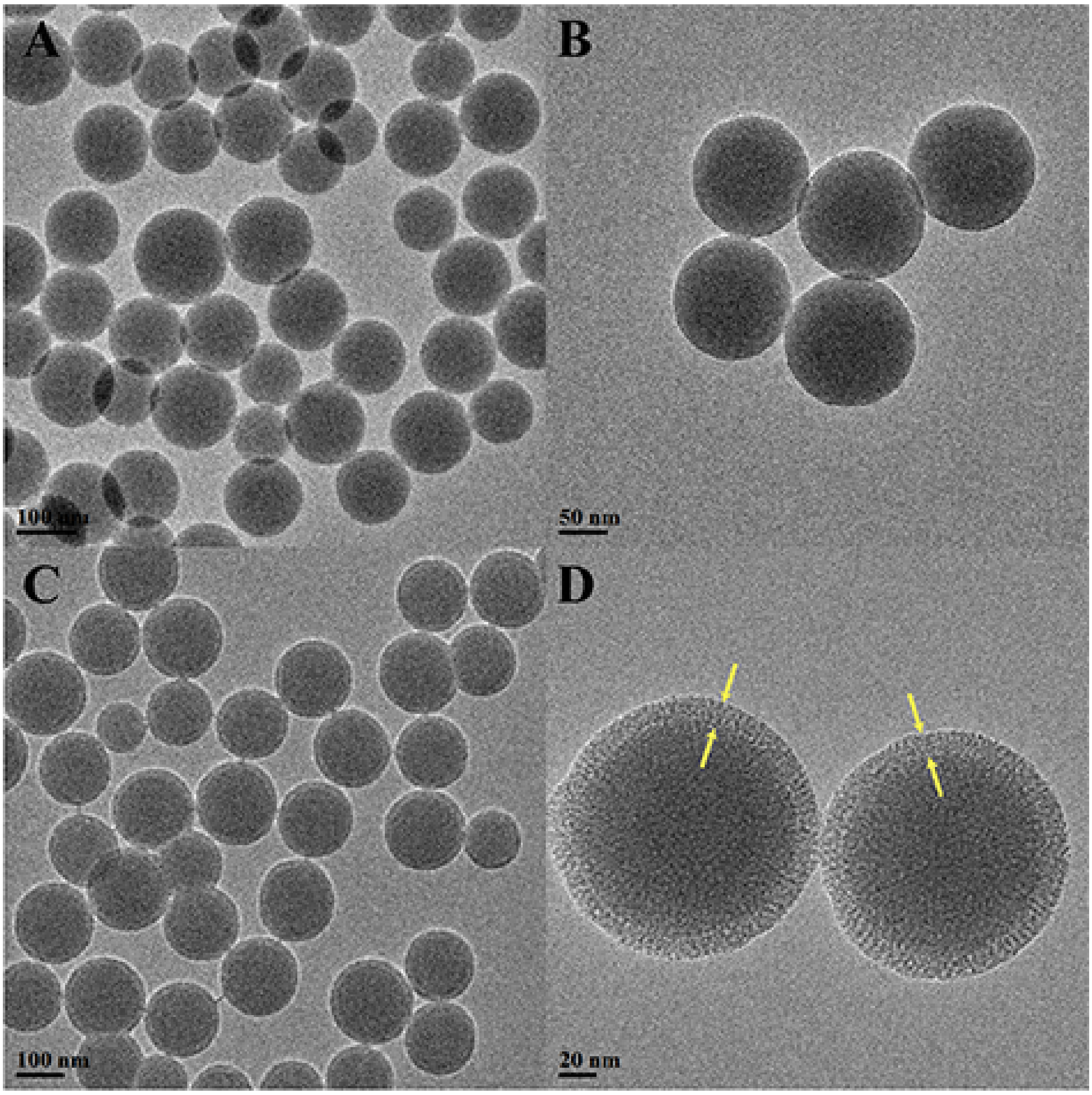

Figure S1. TEM images of dense silica nanoparticles (A), (B) and dense silica nanoparticles coated with mesoporous silica shells (C), (D). Evidently, the mesoporous silica shells were successfully coated on the dense silica nanoparticles .

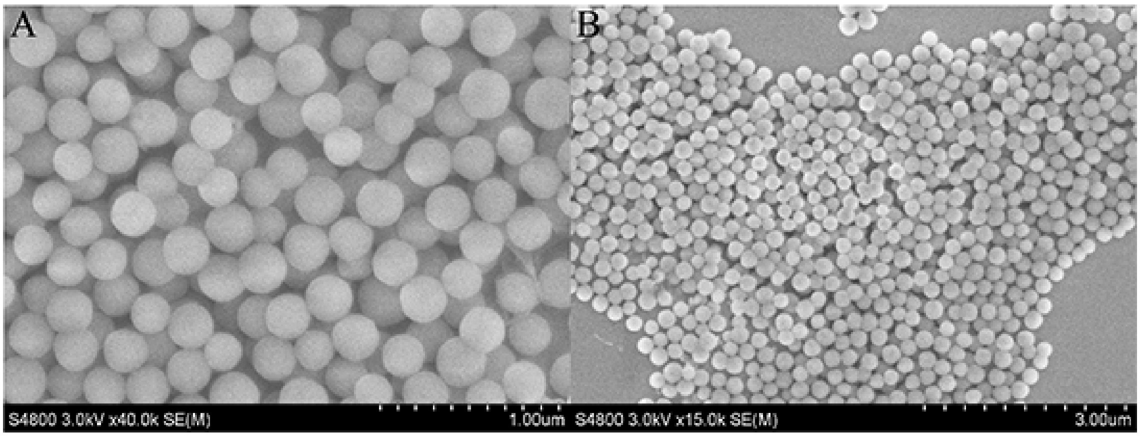

Figure S2. SEM images of dense silica nanoparticles (A) and dense silica nanoparticles coated with mesoporous silica shells (B).

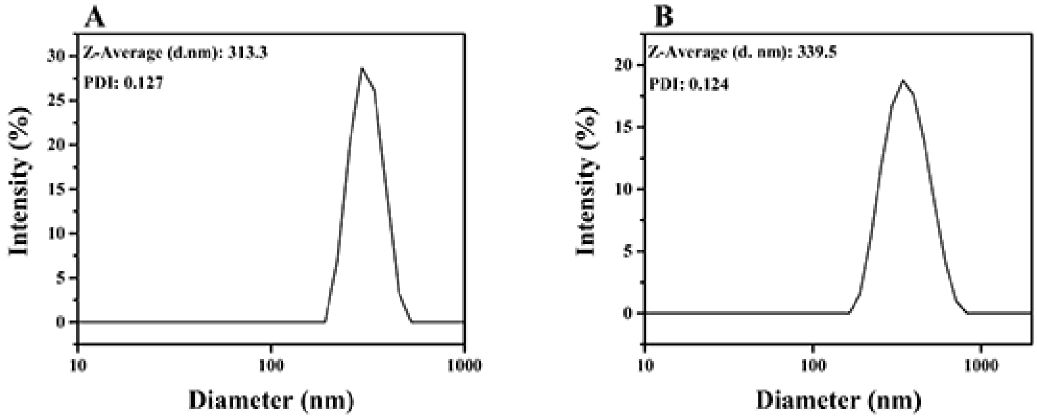

Figure S3. Dynamic light scattering of HMSN (A) and HMSN@AB@PEG (B).

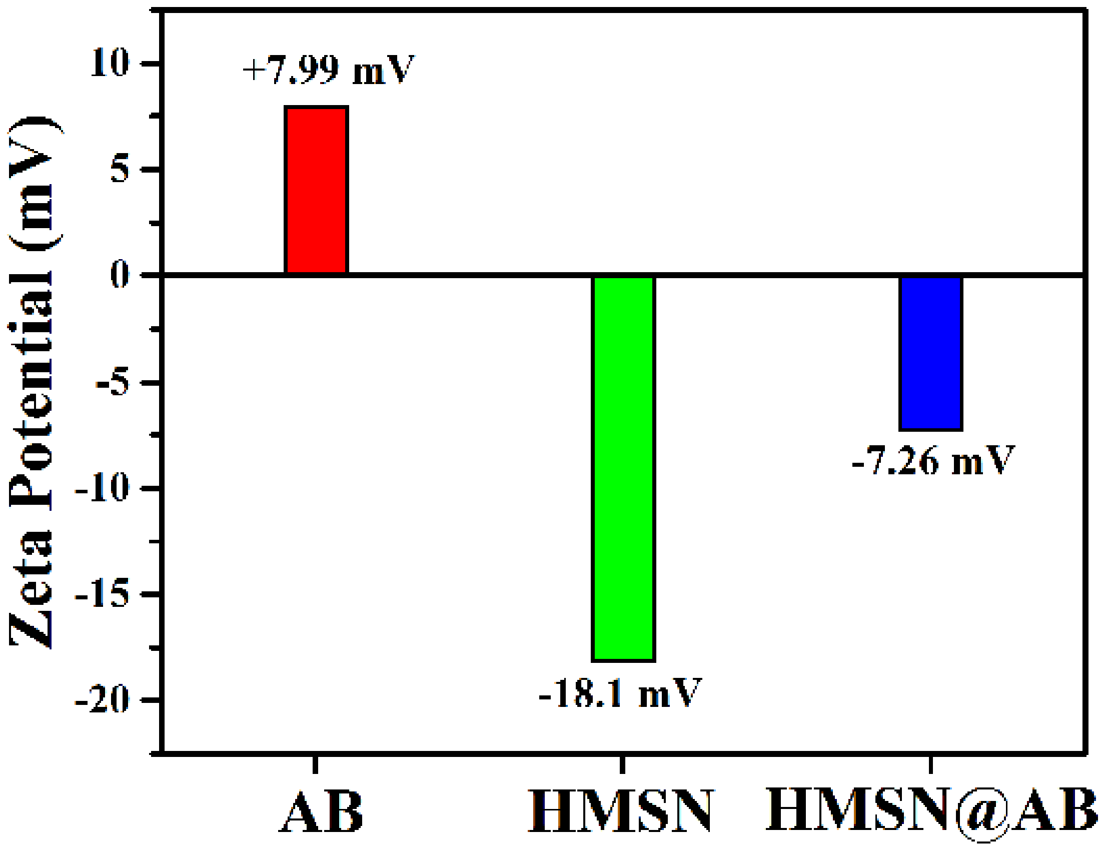

Figure S4. Zeta potential of AB molecules, HMSN nanoparticles and HMSN@AB nanoparticles.

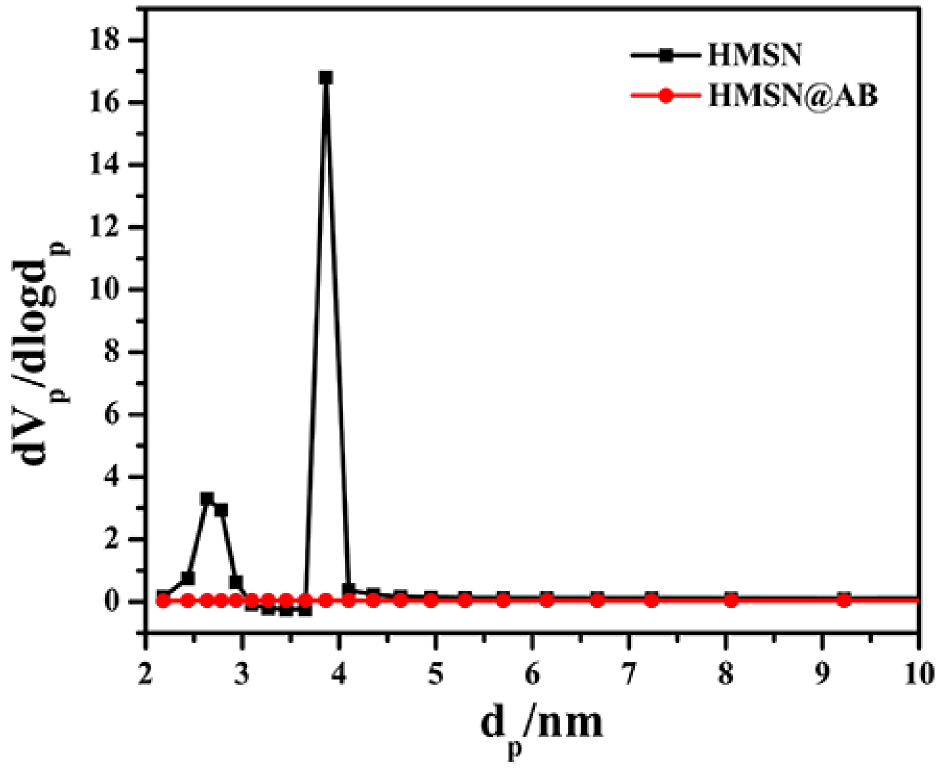

Figure S5. Pore diameter distribution of HMSN and HMSN@AB.

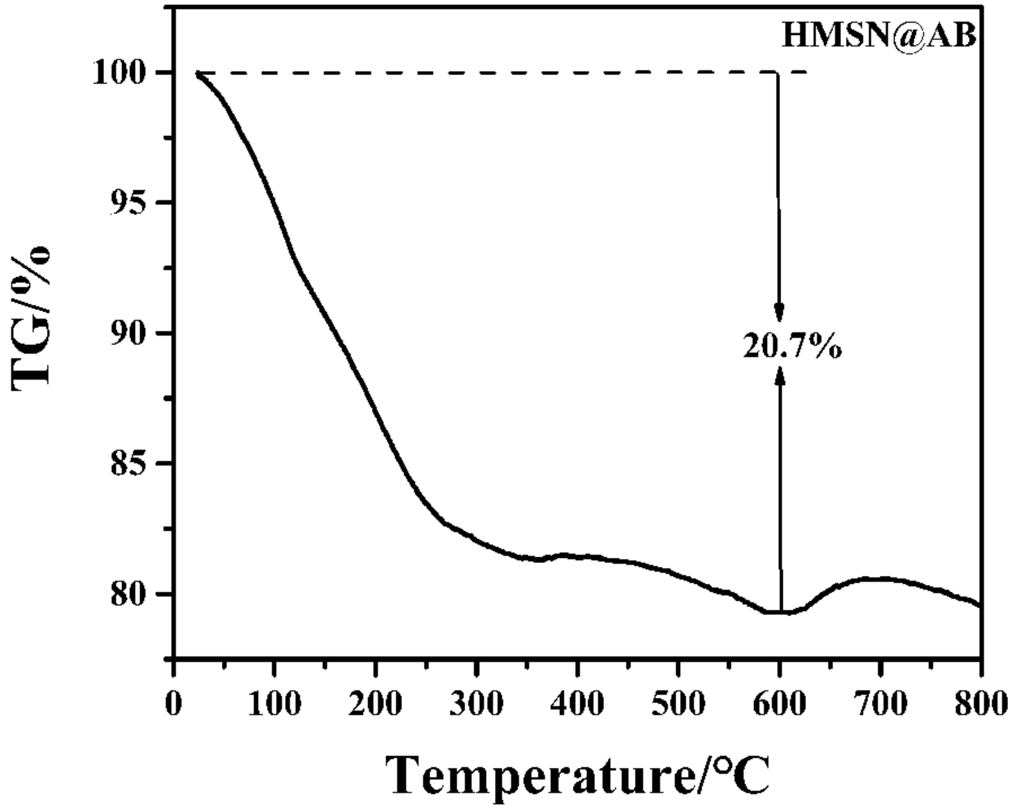

Figure S6. Thermogravimetric analysis curve of HMSN@AB.

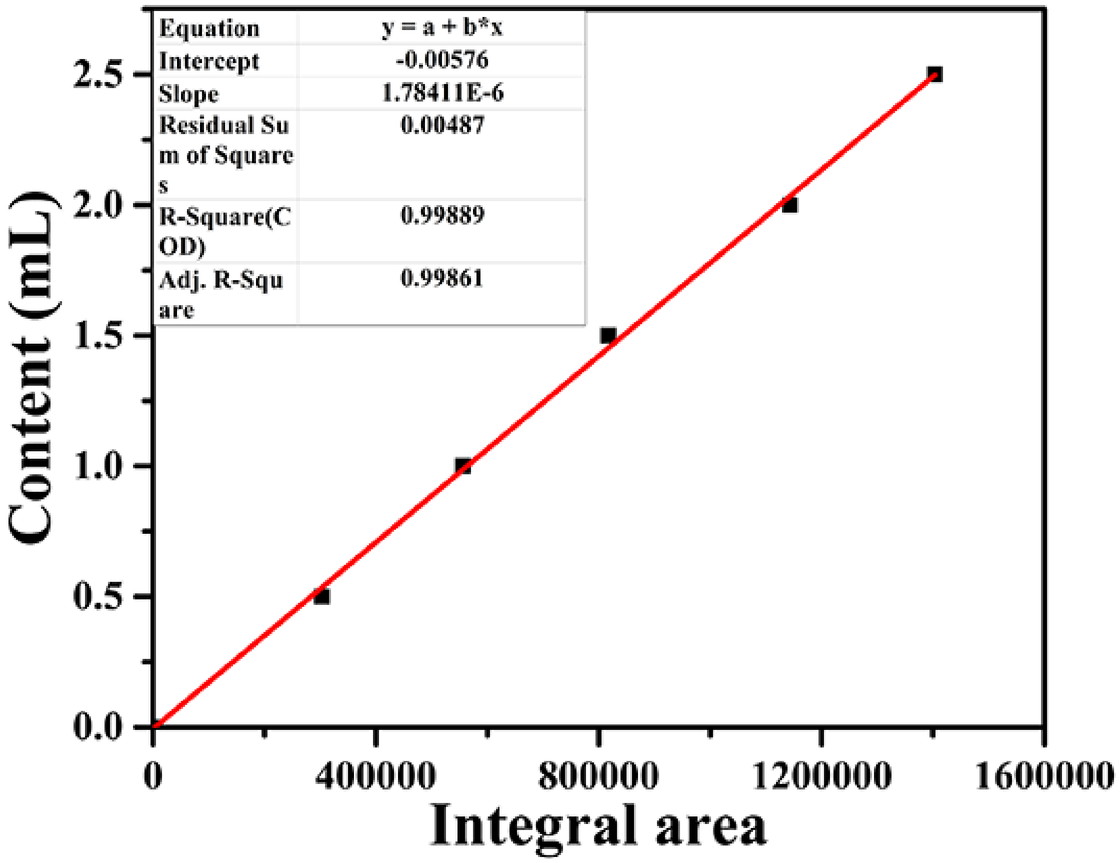

Figure S7. Gas chromatography standard curve of H_2.

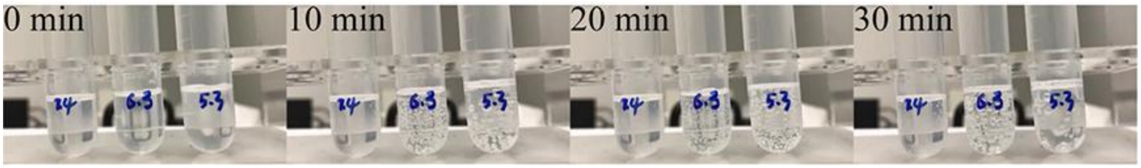

Figure S8. H_2 release process of free AB molecules in different pH (5.3, 6.3, 7.4) during 30 minutes.

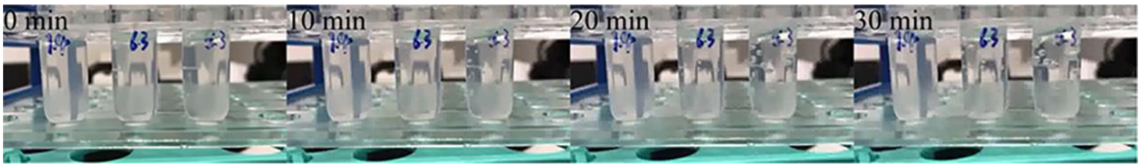

Figure S9. H_2 release process of HMSN@AB@PEG nanoparticles in different pH (5.3, 6.3, 7.4) during 30 minutes.

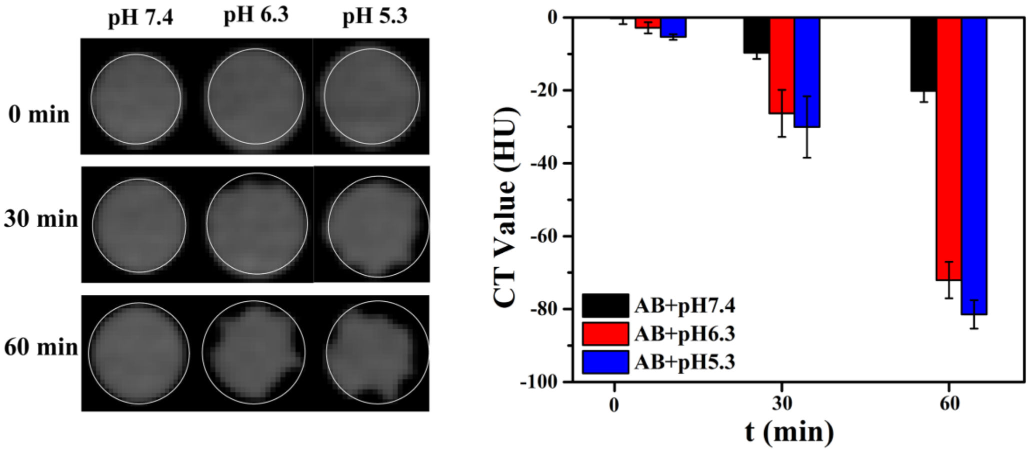

Figure S10. CT images of free AB molecules in different pH (5.3, 6.3, 7.4), the region of white line was the region of interest. And the normalized CT value relative to water of the responding solutions, calculated by the average HU value of 8 slices, which showed the ability of AB molecules as H_2 gas donors to construct a kind of CT gas contrast agent.

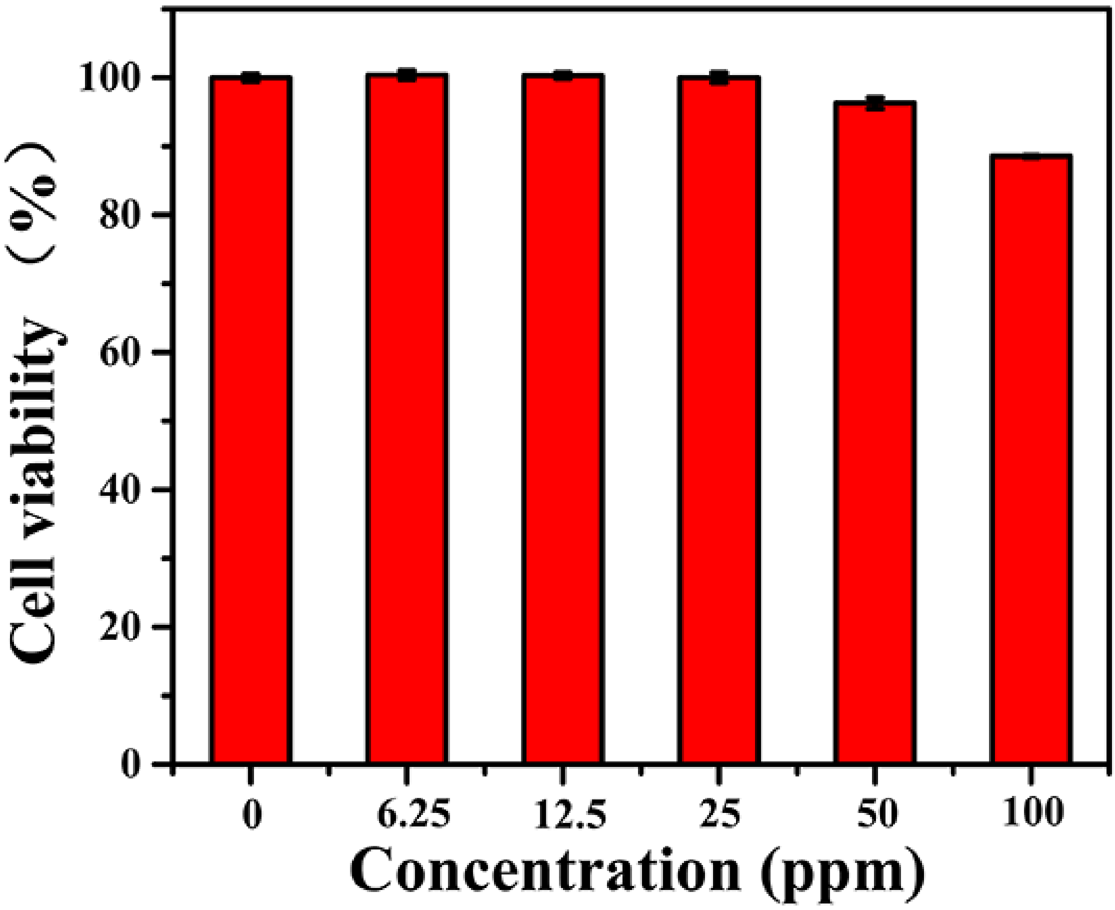

Figure S11. MG63 cells viability of HMSN@AB@PEG nanoparticles with different concentrations (0-100 ppm).

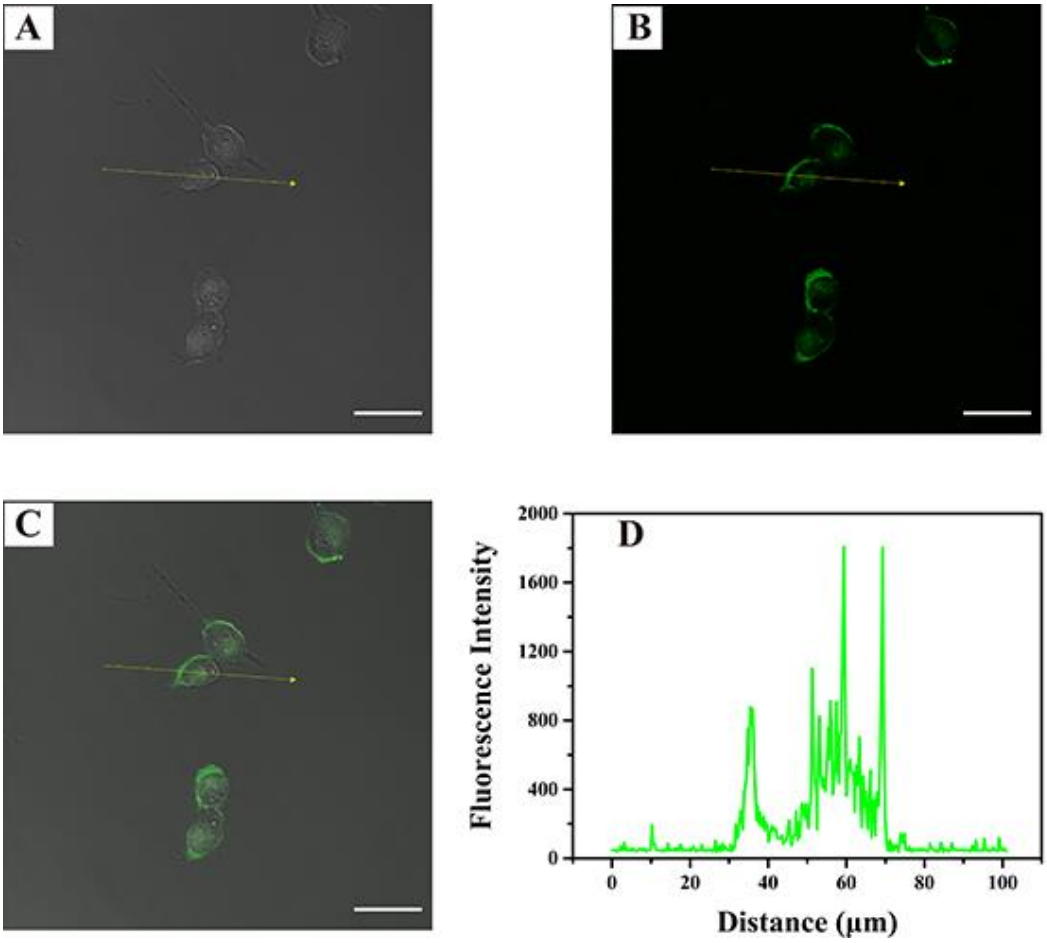

Figure S12. Confocal fluorescence images of MG63 cells incubated with HMSN@AB@PEG nanoparticles, bright field image (A), fluorescence field image (B), overlay image (C); the quantization line of FITC fluorescence intensity (D).

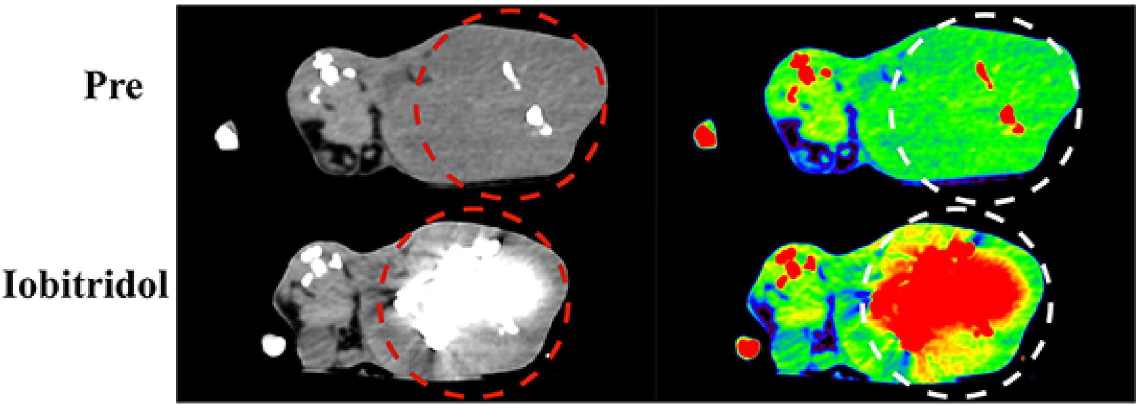

Figure S13. CT imagings (transvers) of osteosarcoma-bearing rats before and after intratumor injection of Iobitridol. The region of dashed line was the area of osteosarcoma. Apparently, from the gray and pseudo-color images, we hardly distinguished osteosarcoma from bones after intratumor injection of Iobitridol. FOV: 350*350 mm, W/L: 300/40.

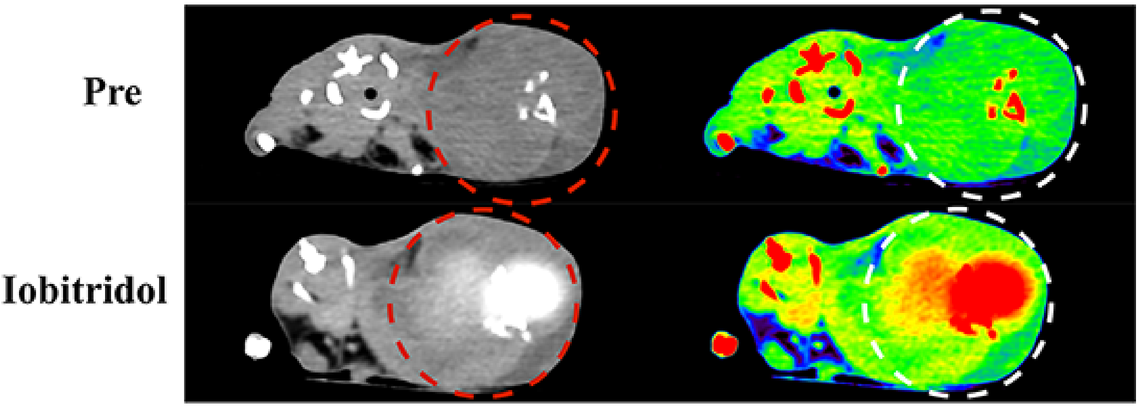

Figure S14. CT imaging (transvers) of osteosarcoma-bearing rats before and after intravenous injection of Iobitridol. The region of dashed line was the area of osteosarcoma. Obviously, from the gray and pseudo-color images, we hardly differentiated osteosarcoma from bones after intravenous injection of Iobitridol. FOV: 350*350 mm, W/L: 300/40.

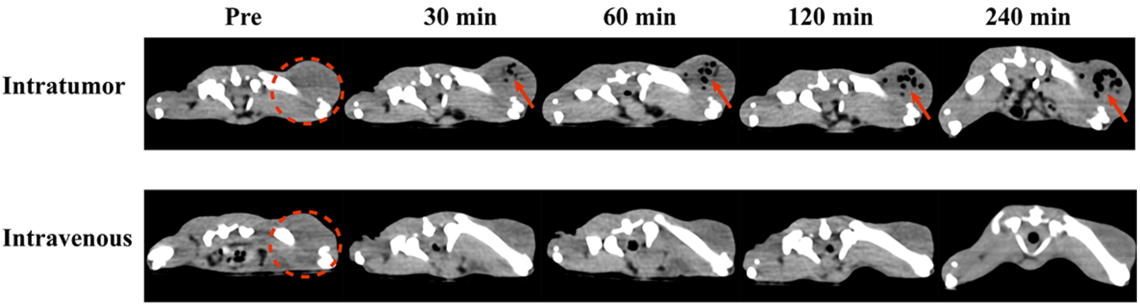

Figure S15. CT imaging (transvers) of osteosarcoma-bearing rats before and after intratumor or intravenous injection of AB molecules (4 mg/mL, 500 μL). FOV: 350*350 mm, W/L: 300/40.

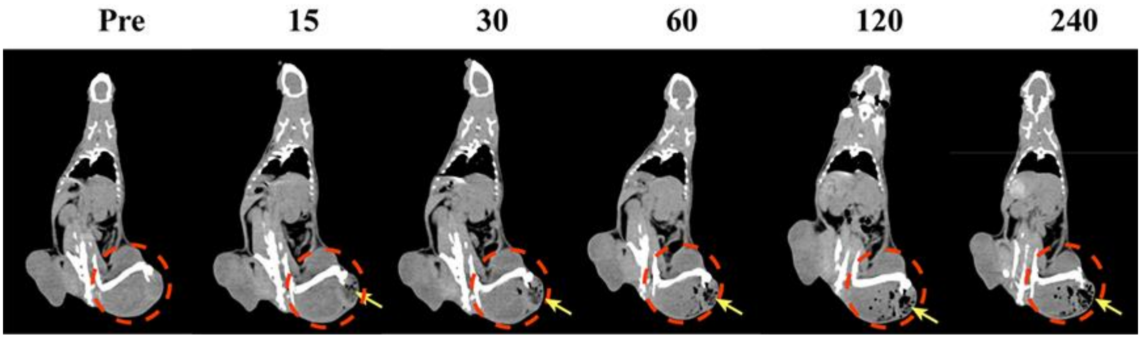

Figure S16. CT gray imagings (coronal) of osteosarcoma-bearing rats before and after intratumor injection of HMSN@AB@PEG (at several time points). The region of dashed line was the area of osteosarcoma, and the region of arrows was the low CT density area of H_2 release. FOV: 350*350 mm, W/L: 300/40.

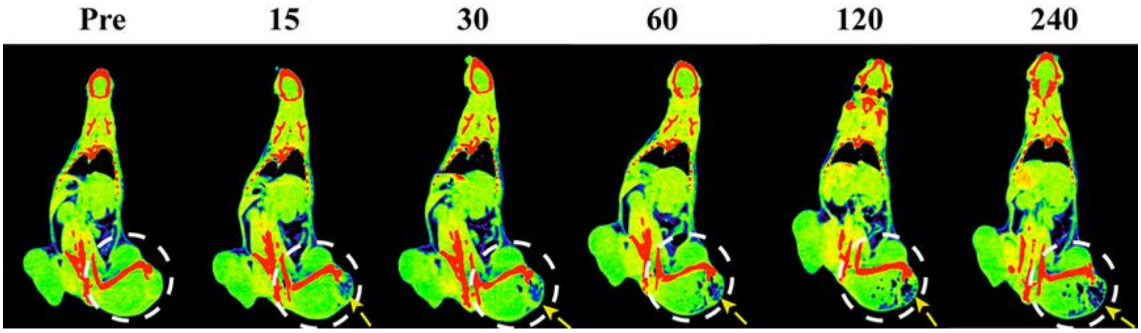

Figure S17. CT pseudo-color imagings (coronal) of osteosarcoma-bearing rats before and after intratumor injection of HMSN@AB@PEG (at several time points). The region of dashed line was the area of osteosarcoma, and the region of arrows was the low CT density area of H_2 release. FOV: 350*350 mm, W/L: 300/40.

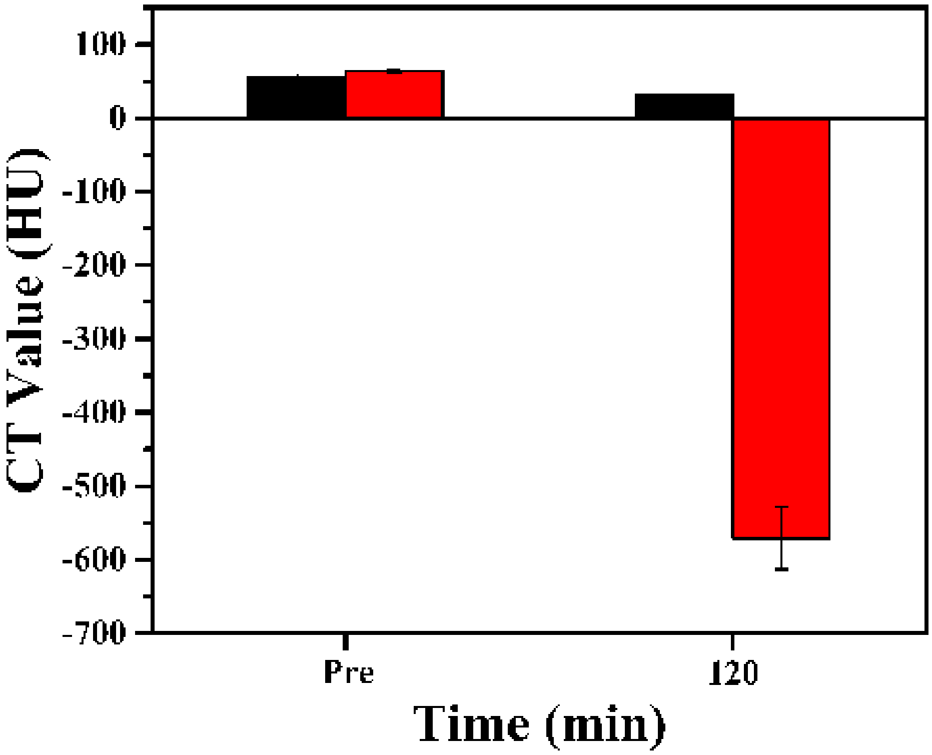

Figure S18. CT density value of two rectangle ROI in gray images before and after intratumor injection of HMSN@AB@PEG (Pre and 120 min).

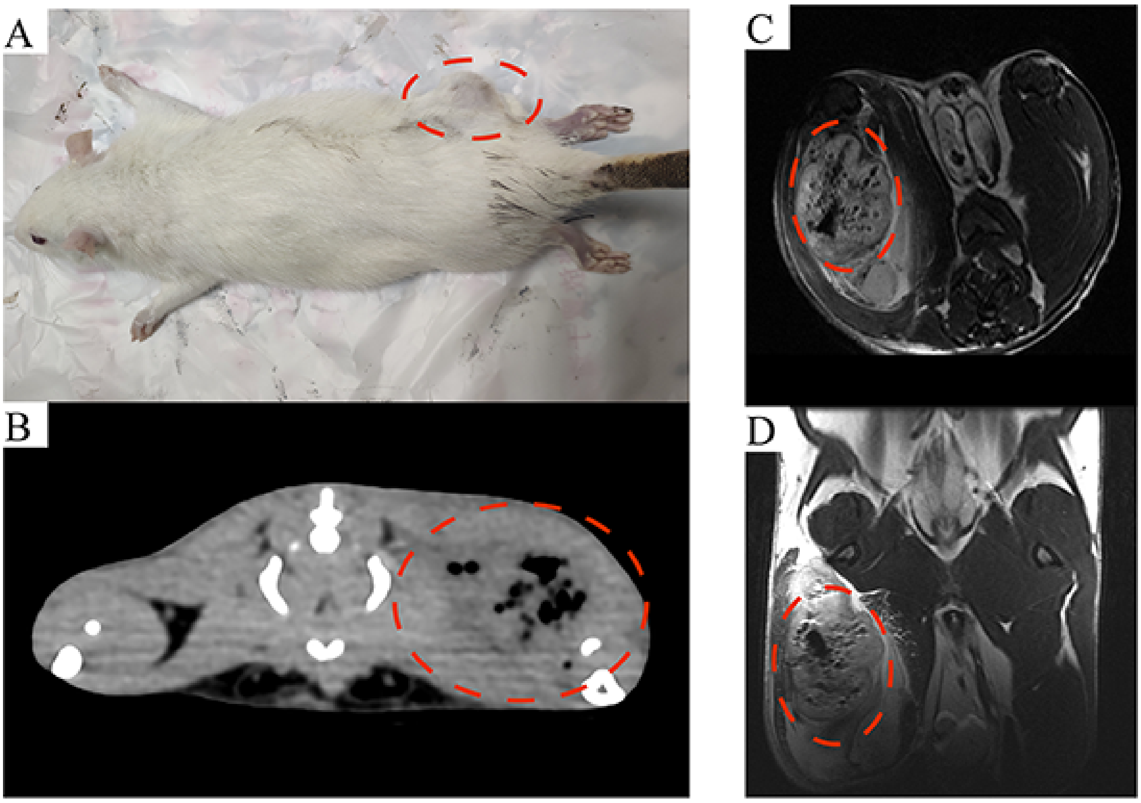

Figure S19. Digital image of osteosarcoma-bearing rat (A); The CT image (B) of osteosarcoma-bearing rat after 240 min intratumor injection of HMSN@AB@PEG, FOV: 244*244 mm, W/L: 300/40; meanwhile, the MRI images (transverse section, C; coronal section, D) were obtained to demonstrated the H_2 produced in the rejoin of osteosarcoma, FOV: 90*90 mm, W/L: 8300/3800, slice thickness: 1.8 mm. The dashed-line region was osteosarcoma.

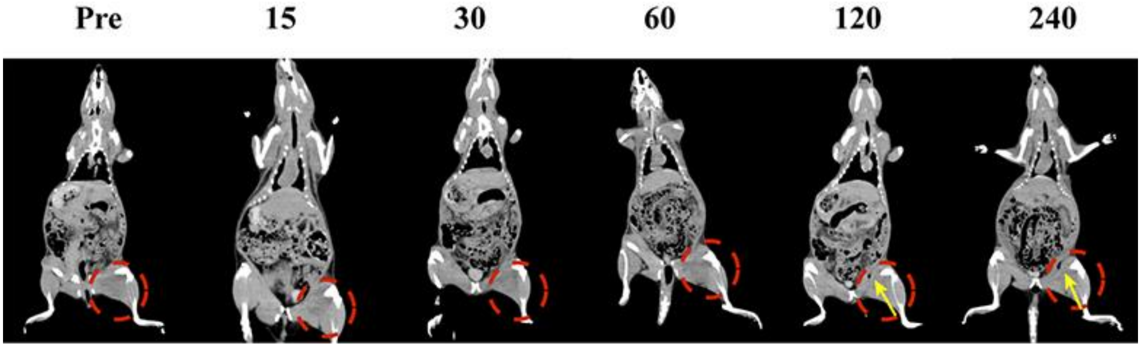

Figure S20. CT gray imagings (coronal) of osteosarcoma-bearing rats before and after intravenous injection of HMSN@AB@PEG (at several time points). The region of dashed line was the area of osteosarcoma, and the region of arrows was the low CT density area of H_2 release. FOV: 350*350 mm, W/L: 300/40.

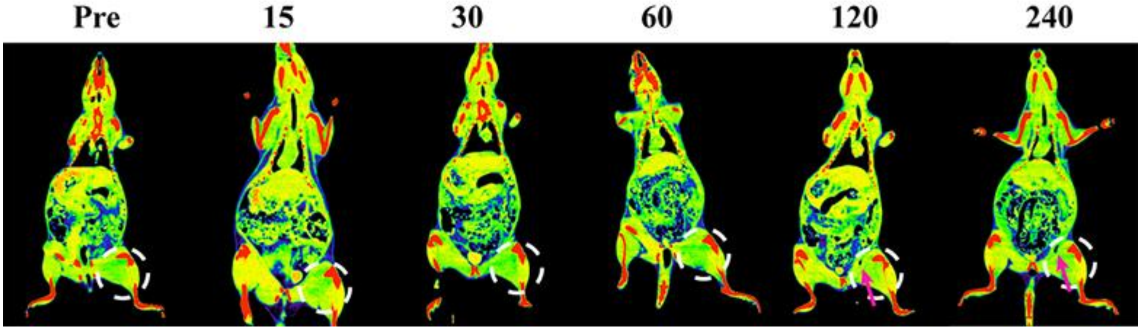

Figure S21. CT pseudo-color imagings (coronal) of osteosarcoma-bearing rats before and after intravenous injection of HMSN@AB@PEG (at several time points). The region of dashed line was the area of osteosarcoma, and the region of arrows was the low CT density area of H_2 release. FOV: 350*350 mm, W/L: 300/40.

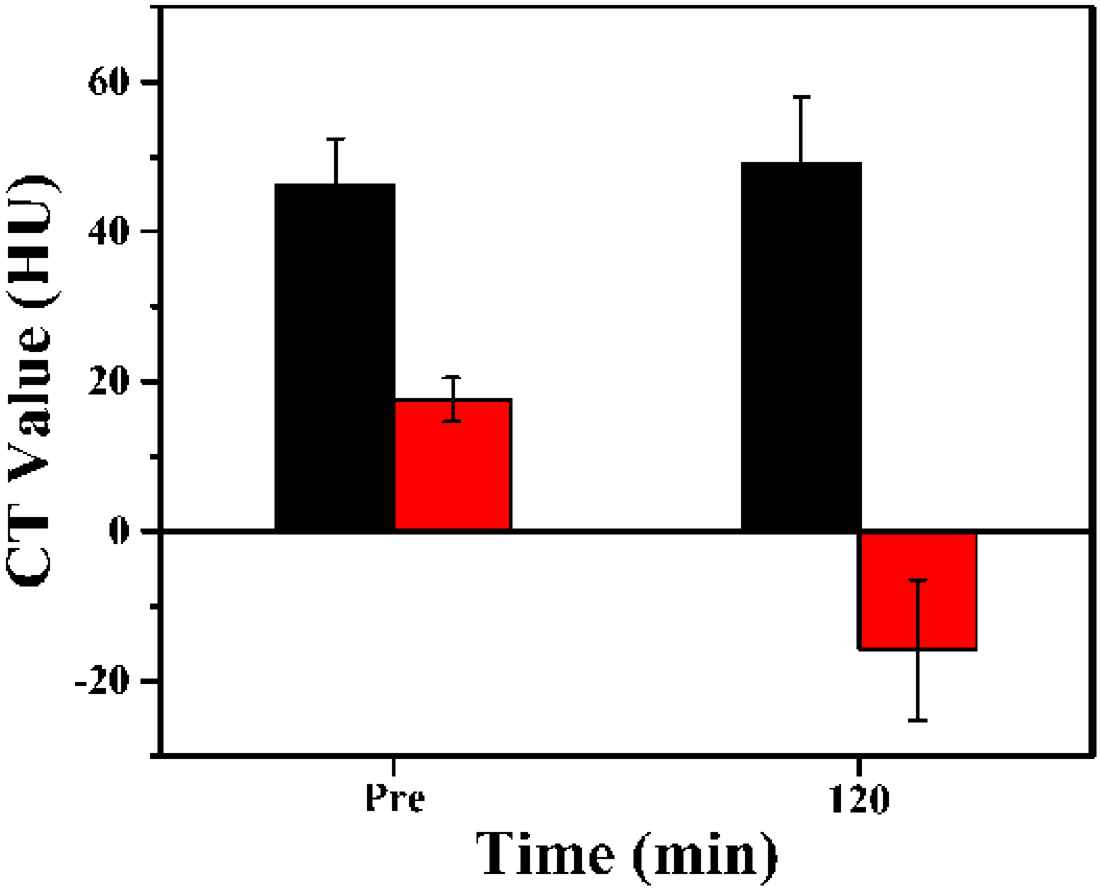

Figure S22. CT density value of two rectangle ROI in gray images before and after intravenous injection of HMSN@AB@PEG (Pre and 120 min).

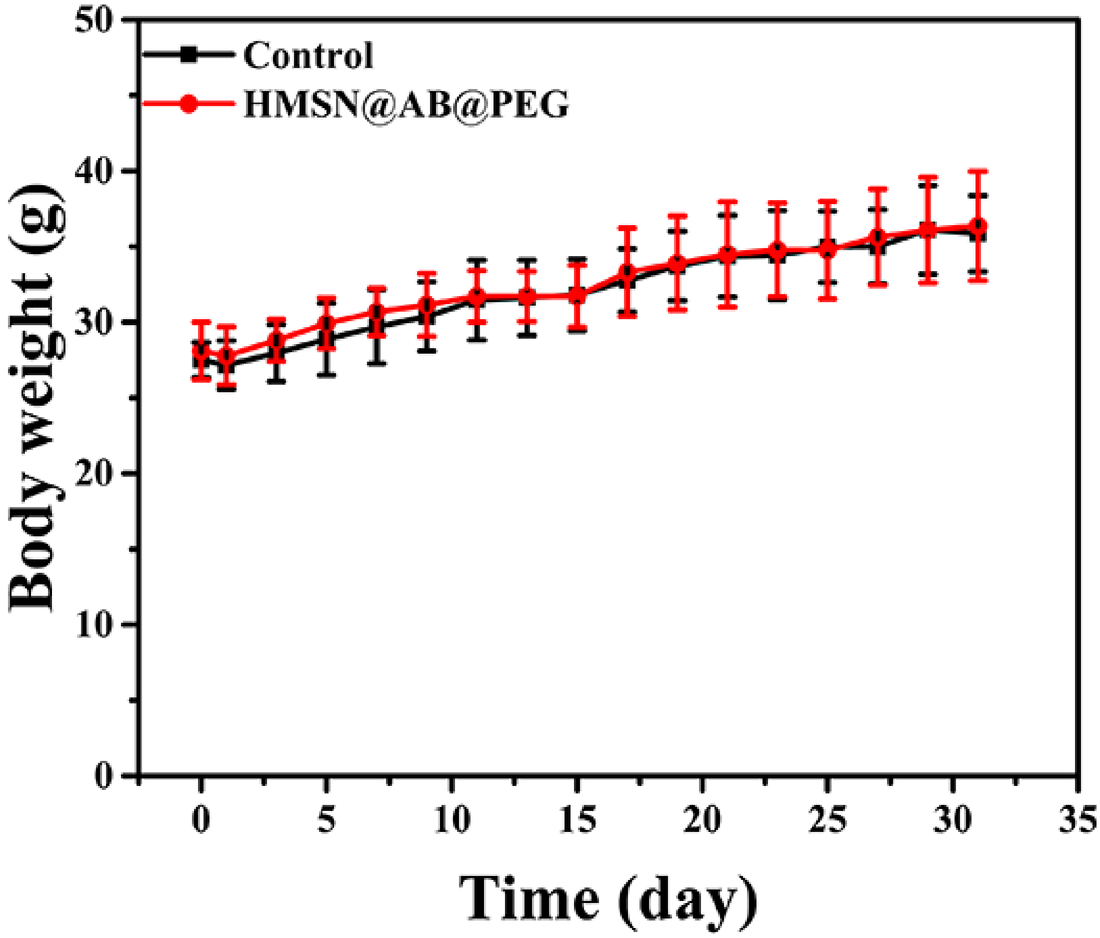

Figure S23. Body weight variation of the experiment groups and control groups during 30 days.

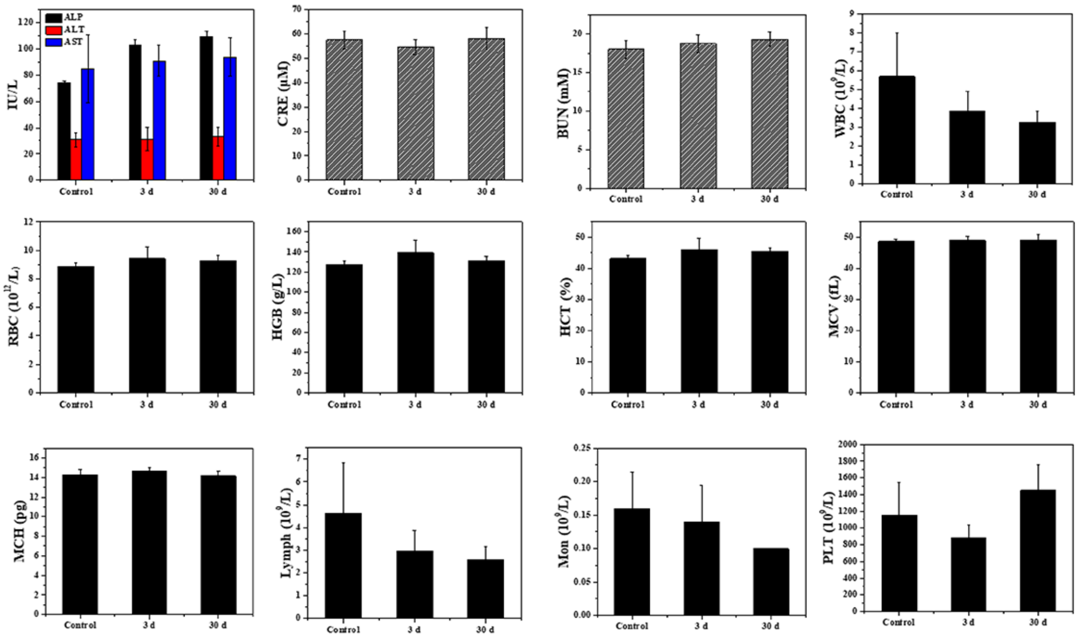

Figure S24. Serum biochemistry and hematology analysis of the experiment groups and control groups.

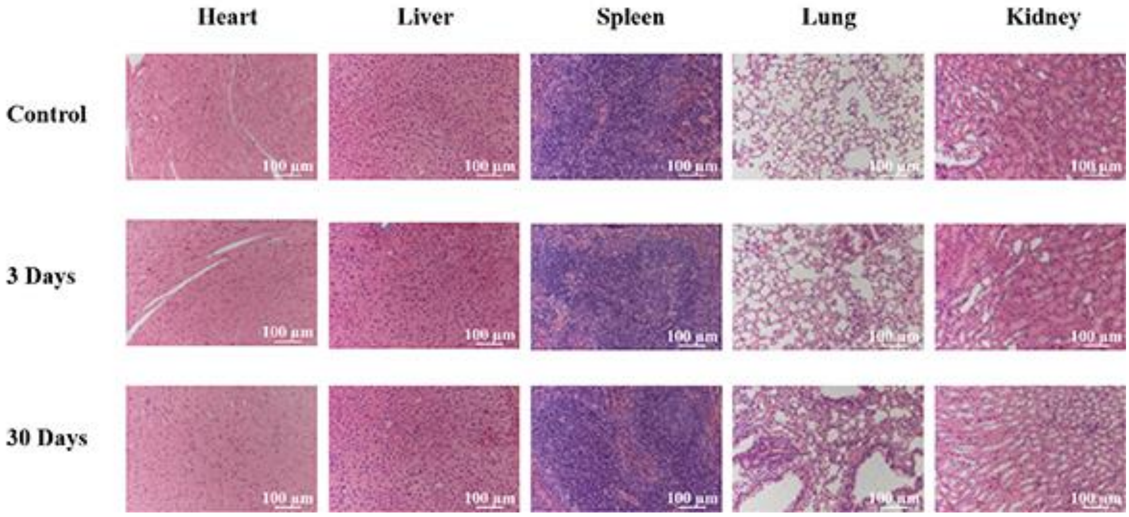

Figure S25. H&E staining analysis of organs including heart, liver, spleen, lung and kidney of the experiment groups and control groups.

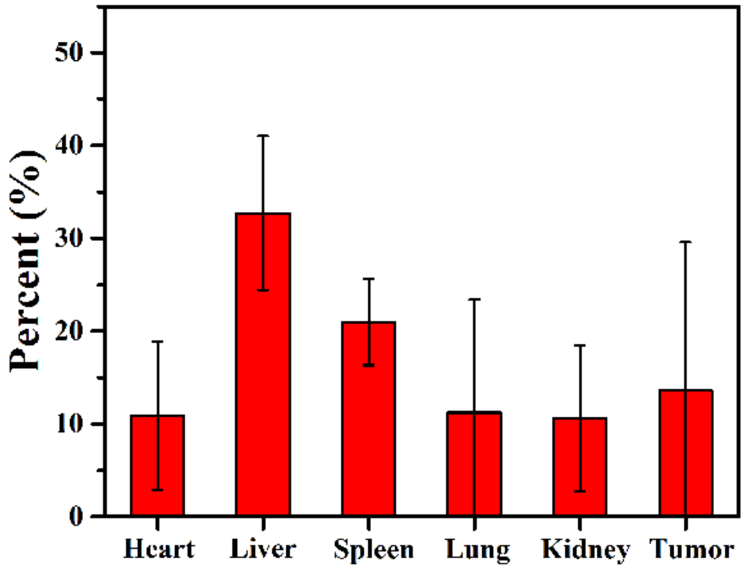

Figure S26. Percentage distribution of Si element in various organs and blood after 4 h intravenous injection of HMSN@AB@PEG.

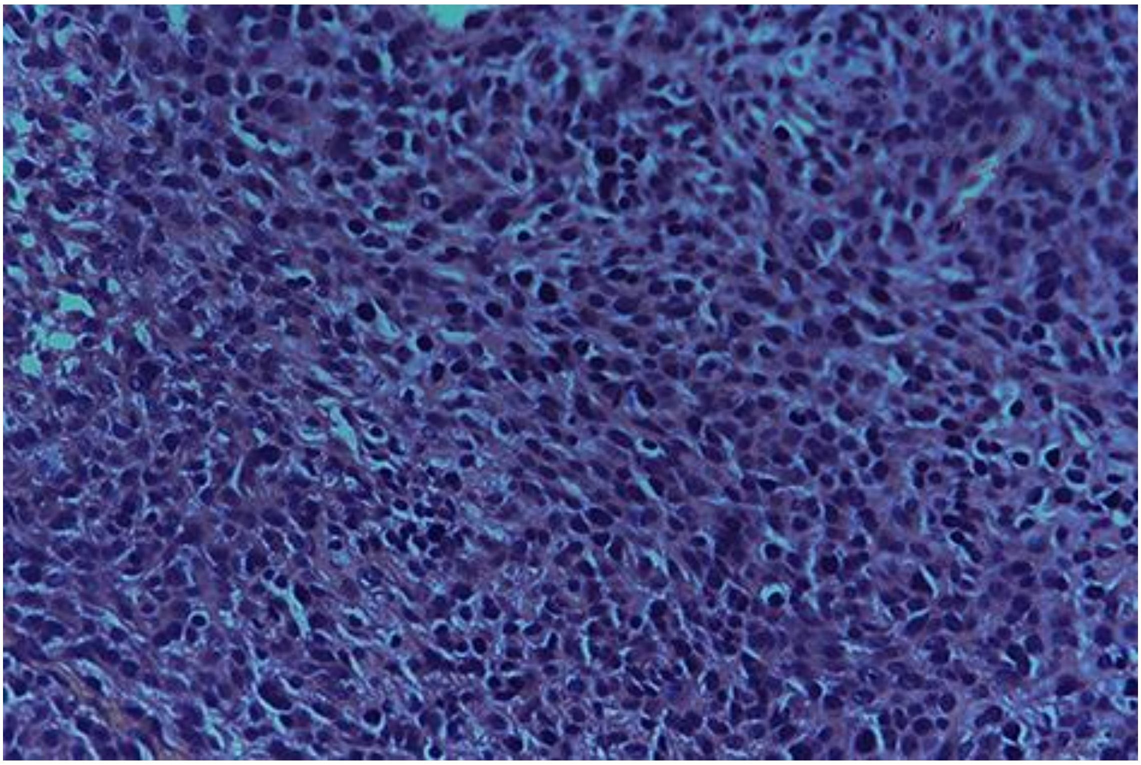

Figure S27. H&E staining image of tumor.

Video S1. H_2 release process of free AB molecules in different pH (5.3, 6.3,7.4) during 30 minutes.

Video S2. H_2 release process of HMSN@AB@PEG nanoparticles in different pH (5.3, 6.3,7.4) during 30 minutes.

Video S3. 3-D video of FITC fluorescence intensity after MG63 cells incubated with HMSN-FITC nanoparticles. The video showed strong fluorescence intensity inner the cells from different angles and levels, which illuminated the nanocomposites possessed good biocompatibility with MG63 cells.

Video S4. CT images of different slices after 4 h intratumor injection of HMSN@AB@PEG nanoparticles in the osteosarcoma.
